# Resuscitation Prior to Emergency Endotracheal Intubation: Results of a National Survey

**DOI:** 10.5811/westjem.2016.6.30503

**Published:** 2016-07-26

**Authors:** Robert S. Green, Dean A. Fergusson, Alexis F. Turgeon, Lauralyn A. McIntyre, George J. Kovacs, Donald E. Griesdale, Ryan Zarychanski, Michael B. Butler, Nelofar Kureshi, Mete Erdogan

**Affiliations:** * Dalhousie University, Department of Critical Care, Halifax, Nova Scotia, Canada; † Trauma Nova Scotia, Halifax, Nova Scotia, Canada; ‡ University of Ottawa, Department of Medicine, Division of Clinical Epidemiology, Ottawa, Ontario, Canada; § University of Ottawa, Ottawa Hospital Research Institute, Clinical Epidemiology Program, Ottawa, Ontario, Canada; ¶ Université Laval, CHU de Quebec Research Center, Hôpital de l’Enfant-Jesus, Population Health and Optimal Health Practices Unit, Trauma-Emergency-Critical Care Medicine Group, Department of Anesthesiology and Critical Care Medicine, Division of Critical Care Medicine, Quebec City, Quebec, Canada; || University of Ottawa, Department of Medicine, Division of Critical Care Medicine, Ottawa, Ontario, Canada; # Dalhousie University, Department of Emergency Medicine, Halifax, Nova Scotia, Canada; ** University of British Columbia, Department of Anesthesia, Pharmacology and Therapeutics, Vancouver, Department of Medicine, Division of Critical Care, Vancouver, British Columbia, Canada; †† Vancouver Coastal Health Research Institute, Centre for Clinical Epidemiology and Evaluation, Vancouver, British Columbia, Canada; ‡‡ CancerCare Manitoba, Department of Haematology and Medical Oncology, Winnipeg, Manitoba, Canada; §§ University of Manitoba, Winnipeg Regional Health Authority, George & Fay Yee Center for Healthcare Innovation, Department of Internal Medicine, Winnipeg, Manitoba, Canada

## Abstract

**Introduction:**

Respiratory failure is a common problem in emergency medicine (EM) and critical care medicine (CCM). However, little is known about the resuscitation of critically ill patients prior to emergency endotracheal intubation (EETI). Our aim was to describe the resuscitation practices of EM and CCM physicians prior to EETI.

**Methods:**

A cross-sectional survey was developed and tested for content validity and retest reliability by members of the Canadian Critical Care Trials Group. The questionnaire was distributed to all EM and CCM physician members of three national organizations. Using three clinical scenarios (trauma, pneumonia, congestive heart failure), we assessed physician preferences for use and types of fluid and vasopressor medication in pre-EETI resuscitation of critically ill patients.

**Results:**

In total, 1,758 physicians were surveyed (response rate 50.2%, 882/1,758). Overall, physicians would perform pre-EETI resuscitation using either fluids or vasopressors in 54% (1,193/2,203) of cases. Most physicians would “always/often” administer intravenous fluid pre-EETI in the three clinical scenarios (81%, 1,484/1,830). Crystalloids were the most common fluid physicians would “always/often” administer in congestive heart failure (EM 43%; CCM 44%), pneumonia (EM 97%; CCM 95%) and trauma (EM 96%; CCM 96%). Pre-EETI resuscitation using vasopressors was uncommon (4.9%). Training in CCM was associated with performing pre-EETI resuscitation (odds ratio, 2.20; 95% CI, [1.44–3.36], p<0.001).

**Conclusion:**

Pre-EETI resuscitation is common among Canadian EM and CCM physicians. Most physicians use crystalloids pre-EETI as a resuscitation fluid, while few would give vasopressors. Physicians with CCM training were more likely to perform pre-EETI resuscitation.

## INTRODUCTION

Resuscitation of critically ill patients often commences with emergency endotracheal intubation (EETI) and the institution of mechanical ventilation, which is commonly performed by emergency medicine (EM) and critical care medicine (CCM) physicians.^1^ Unfortunately, adverse events related to EETI, such as failed intubation, hypoxemia, and post-intubation hypotension, occur at an increased rate compared to elective endotracheal intubations.^2^,^3^ Hemodynamic instability in the peri-intubation phase can result from a variety of factors such as loss of sympathetic drive with medications, positive pressure ventilation, diverse adverse effects of medications, skill level of the intubator, availability of resources, and the patient’s physiology, illness and comorbidities.^4^,^5^ Patient optimization prior to EETI may be important to ensure hemodynamic stability and adequate oxygen delivery in an attempt to minimize further deterioration during the physiologic challenge of EETI, including post-intubation hypotension and hypoxemia, which are associated with increased morbidity and mortality.^3^,^4^,^6^

Pre-EETI patient resuscitation to optimize hemodynamics commonly involves the use of intravenous (IV) fluids and vasopressor medications; however, there are no standards of care guiding these practices. Most EETI studies have focused on the intubation and post-intubation phases of care.^7^,^8^ Although there is a substantial body of literature regarding pre-oxygenation prior to EETI,^9^–^11^ few publications have addressed the use of fluid and vasopressors for pre-EETI resuscitation. There is some evidence to suggest that pre-EETI fluid administration and vasopressor use reduces the incidence of life-threatening complications associated with intubation in intensive care unit (ICU) patients.^12^ More information is needed on strategies used by physicians to optimize hemodynamics during EETI; this information will be useful in planning future research studies to investigate the effects of pre-EETI strategies on patient outcomes.

The objective of this study was to better understand the practices used by Canadian EM and CCM physicians to optimize hemodynamics in critically ill patients undergoing EETI. Specifically, we sought to evaluate whether physicians use resuscitation fluids and vasopressor medications pre-EETI, and to identify physician characteristics associated with performing pre-EETI resuscitation.

## METHODS

To determine the resuscitation practices of physicians prior to EETI, we developed a clinical scenario-based cross-sectional survey using SelectSurvey (www.selectsurvey.net) and distributed it to Canadian EM and CCM physicians. Face and content validity for the survey was ensured through an iterative process among the authors and the Canadian Critical Care Trials Group (CCCTG). Agreement by all members was required for the final survey version. Information on physician characteristics was collected through the survey including their primary specialty, number of years in practice (post-residency time only), academic affiliation (academic or community hospital), and whether they had completed a fellowship in CCM. The survey was comprised of three clinical scenarios (congestive heart failure [CHF], pneumonia, and trauma). The scenarios and complete survey are available in the Supplementary Materials. Clinicians were asked to answer questions using a five-point Likert scale (always, often, sometimes, rarely, never) based on “what they would do if they were managing the scenario in their usual place of work” to allow for potential variation in respondent practice, support, and resources. The Institutional Research Ethics Board approved this study.

We used a combined web-based and postal survey strategy. The questionnaire was distributed via email (with an electronic link to SelectSurvey) and mailed (along with a pre-stamped envelope) to all EM and CCM physician members of the Canadian Association of Emergency Physicians (CAEP), the Canadian Critical Care Society (CCCS) and the CCCTG. Membership lists were combined and any duplicate names, physical addresses, or email addresses were identified and removed. The survey was administered in both English and French. After the initial email distribution, physicians who did not return their survey were sent an additional email survey four weeks after the first mailing. This process was repeated, and non-responders from this third email were then mailed a paper copy of the survey to return by post. Controls were put in place that allowed each physician to only complete the survey one time, and respondent anonymity was assured by coordination of survey distribution by a blinded administrative support person. Study participation was voluntary and completion of any part of the survey implied consent to participate.

The study definition of pre-EETI was determined through consensus among members of the research team and experts in EM and CCM. A respondent was defined as practicing pre-EETI resuscitation if they met either of the following two criteria: a) they answered “always” or “often” to whether they would give the patient IV fluid during the pre-EETI phase and, depending on the type, also gave a specific amount “always” or “often” (crystalloids, > 1000mL; 5% albumin, > 500mL; 25% albumin, any volume; packed red blood cells, any volume; synthetic colloids, > 500mL); or b) they “always” or “often” would give a vasopressor in the pre-EETI phase, and if they did, would administer it via a peripheral venous catheter (i.e., would not delay resuscitation while a central venous catheter was inserted). Internet-based survey responses (via the electronic survey tool) were used to populate the database directly as per design. Paper mail survey responses were manually entered into the electronic database. The accuracy of survey data quality control was assured by performing a double check of 10% of the paper survey responses. We used data from completed survey questions; questions without a response were not included in the analysis. We collected data on physician characteristics (primary specialty, number of years in practice, clinical workload, and academic affiliation) as well as their stated pre-EETI practices (intravascular solutions, vasopressor medications, and methods for obtaining vascular access).

Respondents were grouped by specialty as either an EM or a CCM physician. The EM group included the specialties EM (FRCPC [Fellow of the Royal College of Physicians of Canada]), EM (CCFP-EM [Canadian College of Family Physicians – EM certificate]), EM (CCFP or other), and family medicine; the CCM group was limited to intensivists and included physicians from the specialties of internal medicine, anesthesia, surgery, as well as any physician who had completed a CCM fellowship. We described physician characteristics and self-reported pre-EETI practices using proportions. Reported monitoring parameters and resuscitation end-points were graphically represented as diverging stacked bar charts using a compressed five-point Likert scale (always/often, sometimes, and rarely/never). We used multivariable logistic regression to model the association between predictor variables of physician characteristics (primary specialty [reference: internal medicine], years of practice [reference: < 1 year], CCM fellowship [reference: no CCM fellowship]) and dichotomous outcome variables (practicing pre-EETI resuscitation). Associations that were identified through the multivariable analyses were expressed as odds ratios (ORs) and 95% confidence intervals (CIs). For all statistical tests, a p-value of < 0.05 was considered to be significant. We performed all analyses using IBM SPSS Statistics Version 21 and R (version 3.1.0, Spring Dance) in the RStudio GUI (version 0.98.932).^13^

## RESULTS

### Characteristics of Respondents

The response rate to the survey was 50.2% (882/1758). A quality check of responses from 10% of paper surveys found data in the database to be 99.8% accurate. Table 1 shows characteristics of respondents and includes brief descriptions of physician specialties. When asked about their primary specialty, 72% (634/882) of respondents provided information; 73% (463/634) were grouped as EM physicians and 27% (171/634) were grouped as CCM physicians. Among physicians who indicated their level of experience and affiliation, the majority (61%, 403/662) had at least 10 years in practice, with 27% (180/662) reporting over 20 years of experience, and most (79%, 521/661) worked in an academic institution. Twenty-six percent (26%, 171/657) of physicians reported they had completed a CCM fellowship.

### Pre-EETI Resuscitation

Applying the definition of pre-EETI resuscitation developed by the research team, we assessed how many physicians would perform resuscitation prior to EETI in each of the clinical scenarios. Based on their responses, physicians would commonly perform pre-EETI resuscitation in the pneumonia (61%, 508/834) and trauma (83%, 561/675) scenarios, but not in the CHF scenario (18%, 124/694). Of respondents who would perform pre-EETI resuscitation, physicians preferred to administer fluids in the pneumonia (fluids 93%, 472/508; vasopressors 7%, 36/508) and trauma (fluids 98%, 550/561; vasopressors 2%, 11/561) scenarios. In contrast, pre-EETI resuscitation using vasopressors was more common in the CHF scenario (fluids 15%, 19/124; vasopressors 85%, 105/124). Overall, physicians would perform pre-EETI resuscitation using either fluids or vasopressors in 54% (1193/2203) of cases. Physicians chose to administer both fluids and vasopressors as part of their pre-EETI resuscitation strategy in 12% (138/1193) of cases.

### Fluid Resuscitation Prior to EETI

The vast majority of EM and CCM physicians indicated they would “always/often” administer fluid pre-EETI in the pneumonia (EM 98%, 442/451; CCM 96%, 161/168) and trauma (EM 98%, 451/460; CCM 99%, 168/169) scenarios. In the CHF scenario, 45% (191/428) of EM physicians and 46% (71/154) of CCM physicians would “always/often” give fluid pre-EETI. Overall, physicians reported they would “always/often” administer an IV fluid prior to EETI in 81% (1484/1830) of cases.

**Figure 1:**
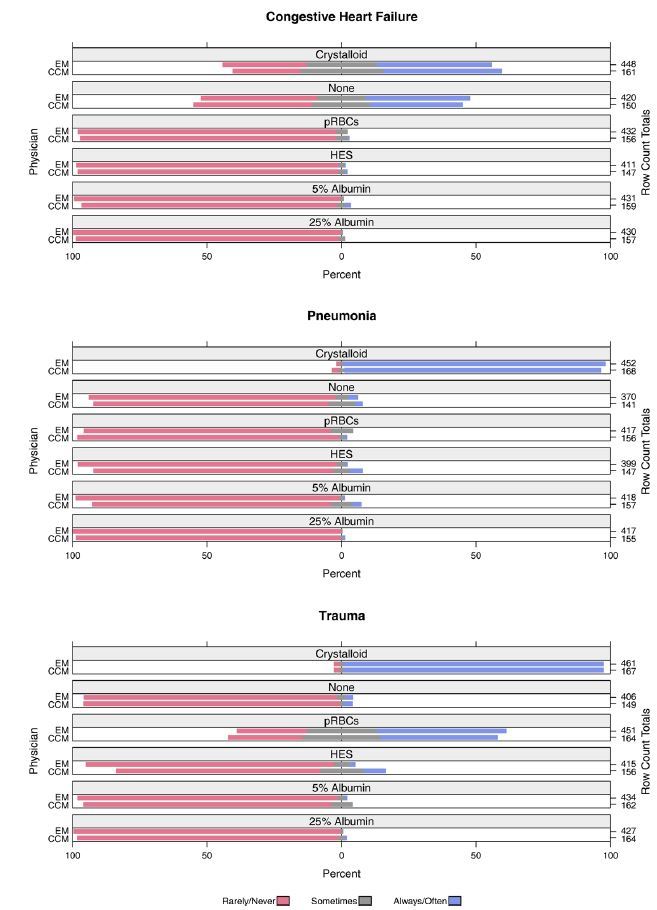
Class of intravenous fluid that emergency medicine (EM) and critical care medicine (CCM) physicians would administer prior to emergency endotracheal intubation in each clinical scenario. *pRBCs*, packed red blood cells; *HES*, hydroxyethyl starch

Preferences of physicians for administering IV fluid prior to EETI are shown in Figure 1. Among IV fluids, a crystalloid fluid (0.9% saline/Lactated Ringer’s) was most frequently selected by physicians as their preference for use (“always/often”) prior to EETI in the CHF (EM 43%, 191/448; CCM 44%, 71/161) pneumonia (EM 97%, 440/452; CCM 95%, 160/168) and trauma (EM 96%, 444/461; CCM 96%, 161/167) scenarios. When asked the approximate volume of IV fluid they normally would administer prior to EETI (based on their preferred type of IV fluid), the amount selected most frequently (“always/often”) was 500–999ml in the pneumonia scenario (53%, 404/760), 1500–1999ml in the trauma scenario (51%, 317/620), and zero fluid (i.e., they would not use any IV fluid) in the CHF scenario (42%, 272/649).

### Pre-EETI Use of Vasopressors

**Figure 2:**
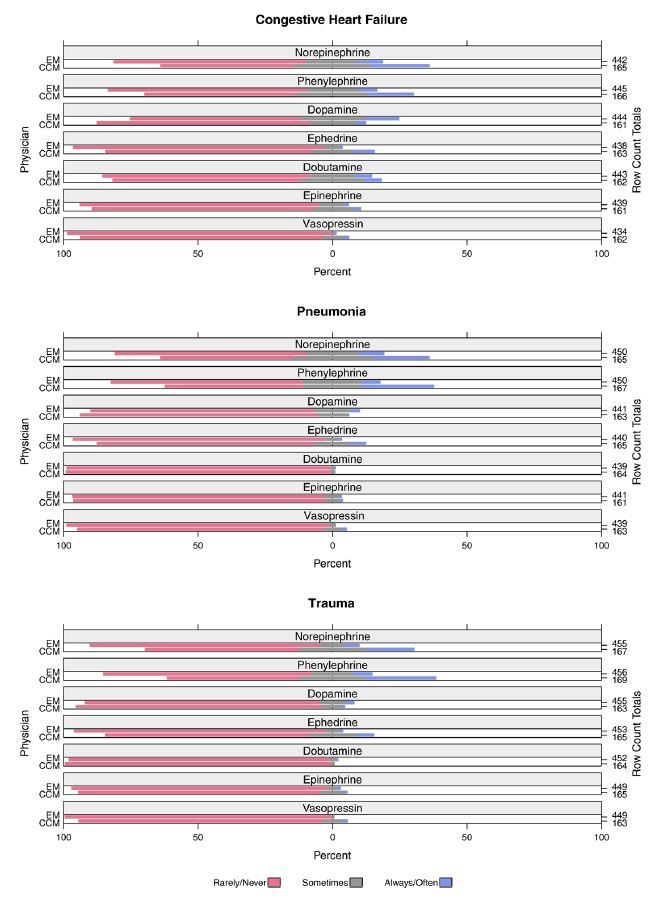
Type of vasopressor that emergency medicine (EM) and critical care medicine (CCM) physicians would normally administer prior to emergency endotracheal intubation (either as a bolus or infusion) in each clinical scenario.

Figure 2 shows physician preferences for how often they would administer various vasopressor medications prior to EETI, assuming the patient in each scenario had no central venous access. Overall, a minority of physicians indicated they would “always/often” use any vasopressors pre-EETI (4.9%, 630/12798). The vasopressors selected most frequently by EM physicians as the ones they would “always/often” administer were dopamine in the CHF scenario (12%, 55/444), norepinephrine in the pneumonia scenario (9%, 42/450), and phenylephrine in the trauma scenario (7%, 32/456). In comparison, the vasopressors CCM physicians indicated they would use “always/often” prior to EETI were phenylephrine in the pneumonia (27%, 45/167) and trauma (26%, 44/169) scenarios, and norepinephrine in the CHF scenario (22%, 36/165). When assessed by physician specialty, CCM physicians were more likely to “always/often” administer a vasopressor prior to EETI (OR, 2.23; 95% CI, [1.91–2.61], p<0.001) compared to EM physicians.

### Vascular Access

In order to establish vascular access for pre-EETI resuscitation, most physicians indicated they would “always/often” use a single peripheral IV in the CHF (EM 75%, 330/440; CCM 73%, 121/166) and pneumonia (EM 77%, 336/435; CCM 75%, 125/167) scenarios. In the trauma scenario, multiple peripheral IVs were chosen most frequently as the method physicians would “always/often” use for obtaining vascular access (EM 95%, 439/463; CCM 90%, 151/168). Overall, most physicians responded they would “rarely/never” insert an arterial catheter (mean 81%, range 79% to 83%) and “rarely/never” insert a central venous catheter (mean 63%, range 54% to 67%).

### Factors Associated with Pre-EETI Resuscitation

A multivariate logistic regression model was fitted to evaluate for association between physician characteristics and whether they would perform resuscitation prior to EETI (Table 2). Physicians who had completed a CCM fellowship were more likely to perform pre-EETI resuscitation (OR, 2.20; 95% CI, [1.44–3.36], p<0.001) compared to physicians without a CCM fellowship. Using physicians with internal medicine specialization as a reference standard, we found that only EM (CCFP-EM or other) training was associated with increased likelihood of providing pre-EETI resuscitation (OR, 1.64; 95% CI, [1.02–2.66], p=0.043) among the specialties of physicians included in the study. There was no association between performing pre-EETI resuscitation and number of years in practice. In comparison with the pneumonia scenario, physicians were significantly more likely to perform pre-EETI resuscitation in the trauma scenario (OR, 1.35; 95% CI, [1.09–1.67], p=0.006) and less likely to practice pre-EETI resuscitation in the CHF scenario (OR, 0.09; 95% CI, [0.07–0.11], p<0.001).

## DISCUSSION

In this pan-Canadian survey of EM and CCM physician practices for optimization of hemodynamics in critically ill patients undergoing EETI, we observed both common practices and practice variability. Using our definition of pre-EETI resuscitation, we found most physicians would perform pre-EETI resuscitation in the scenarios of pneumonia and trauma, but not in CHF. Physicians who would perform pre-EETI resuscitation preferred to use fluids in the pneumonia and trauma scenarios, and vasopressor medications in the CHF scenario. Overall, 81% of physicians indicated they would resuscitate with some amount of IV fluid pre-EETI to optimize patient hemodynamics. Most physicians would administer crystalloid solutions in preparation for EETI, while relatively few would administer a vasopressor medication. The findings of this investigation suggest pre-EETI resuscitation practices vary with patient condition and physician specialty.

Few studies have examined the use of fluids and vasopressors for resuscitation prior to EETI. In a prospective multicenter study of critical care patients, Jaber and colleagues evaluated the implementation of an intubation management protocol that included pre-intubation fluid loading and vasopressor use; they reported significant decreases in both life-threatening (21% vs. 34%, p=0.03) and other complications (9% vs. 21%, p=0.01) in comparison with standard care.^12^ Perbet and colleagues examined the effect of pre-intubation fluid loading as part of a multicenter observational study of risk factors for severe cardiovascular collapse (CVC) after EETI in ICU patients and found it was not significantly associated with the occurrence of CVC.^9^ There is also evidence that implementation of an airway management training program for pulmonary and critical care medicine fellows with emphasis on pre-intubation optimization of hemodynamics, oxygenation, and team/equipment preparation improves the first-attempt success rate for ICU intubations and reduces the rate of complications.^14^ While these studies suggest pre-intubation resuscitation is associated with improved outcomes following EETI, more research is required to elucidate the effects of pre-EETI practices on patient outcomes.

Our findings suggest that the majority of Canadian EM and CCM physicians would resuscitate critically ill patients using crystalloid fluids prior to EETI, yet few physicians would administer vasopressor medications pre-EETI. These results are comparable to an Australian survey of preferences for fluid resuscitation in major trauma patients which found that 85% (56/66) of critical care registrars chose to use crystalloids as a primary resuscitation fluid; however, this study did not specifically investigate resuscitation practices prior to intubation.^15^ Although some studies have surveyed the airway management practices of physicians,^16^–^25^ these have primarily focused on intubation devices, techniques, and medications. Four of these studies examined the practice of rapid sequence intubation in the United Kingdom, the United States, and Canada,^17^, ^23^–^25^ and evaluated whether residents and attending physicians would pre-oxygenate patients before anesthesia induction; these surveys found nearly all respondents (98%–100%) would administer oxygen before induction. None of these studies examined whether physicians or trainees would perform pre-intubation resuscitation using fluids or vasopressors. Our study provides needed information on the pre-EETI resuscitation practices used by physicians to optimize patient hemodynamics.

## LIMITATIONS

The 49.8% survey non-response rate is an important limitation of this study, since non-response bias may affect the validity of our findings. Most of the survey respondents practiced in academic settings; this may bias the results and limit their generalizability to physicians practicing in non-academic settings. Study participants were identified using the mailing lists of CAEP, the CCCS, and the CCCTG. Although these organizations do not represent all EM and CCM physicians in Canada, they were the most comprehensive national listings of EM and CCM physicians that were available to the study team. As with other surveys of physician practices, our results are based on self-reporting rather than documentation or observation, which could introduce information bias and a recall bias. We attempted to minimize bias by instructing physicians to respond based on what they would do if they were managing a patient in their usual place of work.

Another important limitation is that the definition of pre-EETI resuscitation used in this study has not been published previously. To the best of our knowledge, an established definition of pre-EETI resuscitation does not exist. The definition used in this study was developed by the study authors and experts in EM and CCM in an attempt to summarize the available data in a meaningful way. Although we believe our definition reflects common clinical practice, it may not be reflective of all resuscitation practices performed by physicians to optimize patients for EETI. Furthermore, the clinical scenarios developed for this study did not include any description of the volume status of the patients. As conditions such as pulmonary edema and jugular venous distention are likely to impact the strategies used by physicians to treat critically ill patients, the inclusion of information regarding volume status in the scenarios would have strengthened this study. Finally, while we assessed for the association between physician-related factors and performing pre-intubation resuscitation, it is possible we did not consider all potential factors that may contribute to variation in pre-EETI resuscitation practices.

## CONCLUSION

In summary, this study addresses an important phase in the resuscitation of critically ill patients that has not received due attention. The results of this scenario-based survey show that pre-EETI resuscitation using either fluids or vasopressors was provided by physicians in over half (54%) of cases, while both fluids and vasopressors were provided in only 12% of cases. Pre-EETI resuscitation strategies varied with patient condition and physician specialty. Respondents indicated that a crystalloid solution would be administered in many cases prior to EETI, as opposed to vasopressor medications, which were selected in a minority of cases.

## Supplementary Information



## Figures and Tables

**Table 1:** Physician characteristics in study to determine resuscitation practices of physicians prior to emergency endotracheal intubation.

Characteristic	No. (%)
Specialty (n=634)	
EM (CCFP-EM)	275 (43)
EM (FRCPC)	126 (20)
Internal medicine	96 (15)
Anesthesia	57 (9)
EM (CCFP or other)	42 (7)
Family medicine	20 (3)
Surgery	18 (3)
CCM fellowship (n=657)	
No	486 (74)
Yes	171 (26)
Number of years in practice, y (n=662)	
11–20	223 (34)
> 20	180 (27)
6–10	149 (23)
1–5	101 (15)
< 1	9 (1)
Type of practice (n=661)	
Academic	519 (78)
Community	140 (21)
Both	2 (<1)
Currently performing EETI (n=657)	
Yes	651 (99)
No	6 (1)

*EM,* emergency medicine; *CCFP,* Canadian College of Family Physicians; *FRCPC,* Fellow of the Royal College of Physicians Canada; *CCM,* critical care medicine; *EETI*, emergency endotracheal intubation

Values are in n (%).

**Table 2:** Factors associated with pre-emergency endotracheal intubation resuscitation.

Variable	Adjusted OR (95% CI)	p*-*value
Specialty (ref: Internal medicine)		
EM (FRCPC)	1.43 (0.87, 2.33)	0.15
Anesthesia	1.22 (0.77, 1.94)	0.39
EM (CCFP-EM or other)	1.64 (1.02, 2.66)	0.043
Family medicine	1.57 (0.89, 2.76)	0.12
Surgery	0.67 (0.34, 1.34)	0.26
Unknown	0.65 (0.36, 1.15)	0.14
CCM fellowship	2.20 (1.44, 3.36)	<0.001
Years in Practice (ref: < 1 year)		
1–5 years	2.02 (0.80, 5.11)	0.13
6–10 years	1.40 (0.57, 3.49)	0.46
11–20 years	1.24 (0.50, 3.06)	0.64
>20 years	1.11 (0.45, 2.74)	0.83
Unknown	0.43 (0.15, 1.27)	0.13
Scenario (ref: Pneumonia)		
CHF	0.09 (0.07, 0.11)	<0.001
Trauma	1.35 (1.09, 1.67)	0.006

*OR,* odds ratio; *FRCPC,* Fellow of the Royal College of Physicians Canada; *CCFP,* Canadian College of Family Physicians; *EM,* emergency medicine; *CCM,* critical care medicine; *CHF,* congestive heart failure.

Multivariate analysis was adjusted for physician specialty, fellowship in critical care medicine, and number of years in practice.
