# Supplementary material for: Resuscitation Prior to Emergency Endotracheal Intubation: Results of a National Survey
Source: West J Emerg Med. 2016 Jul 26;17(5):542–8. doi: 10.5811/westjem.2016.6.30503 (PMC5017837; doi:10.5811/westjem.2016.6.30503)
Supplement: Supplementary file 1 [file wjem-17-542-s001.pdf]

A Survey of Emergent Endotracheal Intubation in Critically Ill Patients in Canada

Dear Colleagues,

Emergent airway management is an integral part of the resuscitation of critically ill patients, and is routinely performed by Emergency Physicians and Intensivists on a daily basis. Unfortunately, very little is known about how physicians manage patients requiring endotracheal intubation.

As an expert in emergent airway management, we are interested in your approach to intubation, and your views about one particular complication: post-intubation hemodynamic instability (PIHI). On behalf of the Canadian Critical Care Trials Group and the CAEP Critical Care Committee, we ask for your help by completing the following survey.

We appreciate your valuable time, and anticipate that this survey will take approximately 10-15 minutes to complete. If you do not wish to participate, please email me at the address below and we will not contact you again.

If you have any questions regarding the survey, please do not hesitate to contact me at **All information will be protected and is strictly confidential. Results will be reported in aggregate only.**

Thank you in advance for your participation in this project.

Sincerely,

A Survey of Emergent Endotracheal Intubation in Critically Ill Patients in Canada

Scenario 1: Sepsis

You are asked to see a 59-year-old female in the Emergency Department with pneumonia and respiratory distress. Her past history includes hypertension (controlled with a beta blocker, baseline BP 120/70) and hypercholesterolemia.

Her vital signs are HR 120 bpm, RR 32/min, BP 100/56 mmHg, SaO2 90% (on FiO2 100%) and temperature 38.9 C. **You feel that she requires immediate intubation prior to transfer to the ICU. She has no predictors of a difficult airway and her weight is 70 Kg.**

She has no IV access at this point, and has had no medical interventions prior to your arrival. You have the aid of a skilled acute care nurse in addition to an airway cart containing the typical medications and airway equipment available in your practice.

Based on this information, please answer the following questions:

1. **Intravenous access (IV) access**: In a patient similar to the one described in the above scenario (Scenario 1, sepsis), how often would you **insert the following PRIOR** to intubation?

   Please remember that **the patient has no IV access** at the present time.

	Never	Rarely	Sometimes	Often	Always
Single peripheral IV	○	○	○	○	○
Multiple peripheral IV's	○	○	○	○	○
Central venous catheter	○	○	○	○	○
Arterial catheter	○	○	○	○	○

2. **IV fluid administration before emergent endotracheal intubation:** In a patient similar to the one described in the above scenario (Scenario 1, sepsis), please indicate what class of IV fluid (if any) that you would administer **PRIOR** to intubation.

   Please remember that **this patient has not received any IV fluids** prior to this time.

	Never	Rarely	Sometimes	Often	Always
None	○	○	○	○	○
Crystalloid (0.9% saline/Lactated Ringers)	○	○	○	○	○
5% albumin	○	○	○	○	○
25% albumin	○	○	○	○	○
Packed Red Blood Cells	○	○	○	○	○
Synthetic colloid (HES)	○	○	○	○	○

3. **IV FLUID Volume:** In a patient similar to the one described in the above scenario (Scenario 1, sepsis), please indicate the **approximate volume of IV fluid** that you would normally administer **PRIOR** to intubation.

   Please base your answer on the **nost preferred IV fluid as indicated in the question above**.

   -

	Never	Rarely	Sometimes	Often	Always
No IV fluid administration	○	○	○	○	○
<500 ml	○	○	○	○	○
500-999 ml	○	○	○	○	○
1000-1499 ml	○	○	○	○	○
1500-1999 ml	○	○	○	○	○
>2000 ml	○	○	○	○	○

4. **Vasopressor Administration:** In a patient similar to the one described in the above scenario (scenario 1, sepsis), please indicate which (if any) of the following that you would normally administer (**either bolus or infusion**) **PRIOR** to intubation.

   Please note that this patient has not reveived any interventions prior to this time. Her vital signs are HR 120 bpm, RR

32/min, BP 100/56 mmHg, SaO2 90% (on FiO2 100%) and temperature 38.9 C.

	Never	Rarely	Sometimes	Often	Always
phenylephrine	○	○	○	○	○
ephedrine	○	○	○	○	○
dopamine	○	○	○	○	○
norepinephrine	○	○	○	○	○
epinephrine	○	○	○	○	○
dobutamine	○	○	○	○	○
vasopressin	○	○	○	○	○

5. In patients similar to Scenario 1 (sepsis), please indicate how often you would **administer a peripheral vasopressor prior to intubation** (assuming the patient has no central venous access):

	Never	Rarely	Sometimes	Often	Always
ADMINISTER a vasopressor via a PERIPHERAL IV before intubation	○	○	○	○	○
WAIT to administer a vasopressor until after a CENTRAL VENOUS CATHETER is inserted	○	○	○	○	○

A Survey of Emergent Endotracheal Intubation in Critically Ill Patients in Canada

6. In your practice, how often would you use the following medications to facilitate intubation in patients similar to the patient described in the above scenario (Scenario 1, sepsis)?

	Never	Rarely	Sometimes	Often	Always
midazolam	○	○	○	○	○
other benzodiazepine	○	○	○	○	○
fentanyl	○	○	○	○	○
other opioid	○	○	○	○	○
etomidate	○	○	○	○	○
propofol	○	○	○	○	○
ketamine	○	○	○	○	○
succinylcholine	○	○	○	○	○
rocuronium	○	○	○	○	○

7. **Primary intubation strategy**: In a patient similar to the one described in the above scenario (scenario 1, sepsis), **how often would you use the following devices/procedures** to intubate this patient?
-

	Never	Rarely	Sometimes	Often	Always

Curved blade (Macintosh) direct laryngoscope	○	○	○	○	○
Straight blade (Miller) direct laryngoscope	○	○	○	○	○
Bougie assisted direct laryngoscope	○	○	○	○	○
Video laryngoscope (ie. glidescope)	○	○	○	○	○
Other indirect devices (ie. ILMA, optical stylet, airtrac, lightwand, etc.)	○	○	○	○	○
Flexible bronchoscope	○	○	○	○	○
Transtracheal jet ventilation	○	○	○	○	○
Percutaneous wire guided cricothyrotomy	○	○	○	○	○
Open cricothyrotomy	○	○	○	○	○
Tracheostomy	○	○	○	○	○

8. **Secondary/back up intubation strategy**: In the event that **you could not intubate** a patient similar to the patient in Scenario 1, how often would you perform the following?

-

	Never	Rarely	Sometimes	Often	Always
Extraglottic Device (LMA, King LT, etc)	○	○	○	○	○
Trastracheal Jet Ventilation	○	○	○	○	○
Percutaneous Wire Guided Cricothyrotomy	○	○	○	○	○
Open Cricothyrotomy	○	○	○	○	○
Tracheostomy	○	○	○	○	○

A Survey of Emergent Endotracheal Intubation in Critically Ill Patients in Canada

After successful intubation without complication or difficulty, the patient's blood pressure is reduced from a pre-intubation blood pressure of 100/56 mmHg.
For the purposes of the following questions, please assume that the patient's chronic "hypertension" is adequately controlled with outpatient medications, and that she currently has no evidence of other adverse events (ie: SaO2 100%, no myocardial ischemia).

Based on this information, please answer the following information.

9. When considering blood pressure reductions after intubation in patients similar to the patient in scenario 1 (sepsis), what is **the threshold that you would you consider to be "unstable" and actively intervene?**
Select at least 1 and no more than 1.

- ☐ Systolic Blood Pressure <100
- ☐ Systolic Blood Pressure <90
- ☐ Systolic Blood Pressure <80
- ☐ Systolic Blood Pressure <70
- ☐ Systolic Blood Pressure <60
- ☐ Other, please specify

10. When considering blood pressure reductions after intubation in patients similar to the patient in scenario 1 (sepsis), what is **the Mean Arterial Pressure (MAP) threshold that you would actively intervene?**
Select at least 1 and no more than 1.

- ☐ Mean Arterial Pressure <80
- ☐ Mean Arterial Pressure <70
- ☐ Mean Arterial Pressure <60
- ☐ Mean Arterial Pressure <50
- ☐ Mean Arterial Pressure <40
- ☐ Other, please specify

11. Please assume that your **patient meets your threshold for requiring intervention** for hemodynamic instability after emergent intubation. **How long does a patient similar to the patient in scenario 1 (sepsis) have to be hemodynamically unstable before you intervene**?

Select at least 1 and no more than 1.

- ☐ I would treat hemodynamic instability immediately.
- ☐ 1-2 min; I would treat hemodynamic instaility if it has been present for >2 min.
- ☐ 2-4 min; I would treat hemodynamic instability if it has been present for >4 min.
- ☐ 4-6 min; I would treat hemodynamic instability if it has been present for >6 min.
- ☐ 6-8 min; I would treat hemodynamic instability if it has been present >8 min.
- ☐ >10 min; I would treat hemodynamic instability if it has been present >10 min.
- ☐ Never; I would not treat hemodynamic instability.
- ☐ Other, please specify

12. Assuming a patient similar to the patient in Scenario 1 (sepsis) **meets your blood pressure threshold and duration criteria for post-intubation hemodynamic instability**, how would you **manage the patient's hemdynamic instability**?

Please assume that her low blood pressure is the only issue at this time (Oxygen saturation 100%, no evidence of cardiac ischemia or other end-organ dysfunction).

-

	Never	Rarely	Sometimes	Often	Always
Crystalloid (0.9% saline or Lacteted Ringer's) IV fluid administration	○	○	○	○	○
Albumin IV administration	○	○	○	○	○
Synthetic colloid (HES) IV fluid	○	○	○	○	○

administration					
vasopressor administration via a peripheral IV (if no central catheter)	○	○	○	○	○
Vasopressor administration via a central catheter	○	○	○	○	○
I would not treat post-intubation hemodynamic instability	○	○	○	○	○

A Survey of Emergent Endotracheal Intubation in Critically Ill Patients in Canada

Scenario 2: Congestive Heart Failure

You are asked to see a 67-year-old male in the Emergency Department with congestive heart failure and respiratory distress. His past history includes ischemic heart disease (3 previous myocardial infarcts, most recent 8 months ago), ischemic cardiomyopathy (documented ejection fraction 20% 2 months ago), hypertension (controlled with a beta blocker, baseline 120/70), hypercholesterolemia, and mild renal insufficiency (baseline Cr 140 mEq/L).

His vital signs are HR 120 bpm, RR 32/min, BP 100/56 mmHg, SaO2 90% (on FiO@ 100%), and temperature 36.9 C. His EKG demonstrates no acute changes and is similar to his baseline.

You feel that his presentation is due to progression of his ischemic cardiomyopathy, and that he requires immediate intubation. He has no predictors of a difficult airway and his weight is approximately 80 Kg.

He has no IV access at this point, and has had no medical interventions prior to your arrival. You have the aid of a skilled acute care nurse in addition to an airway cart containing the typical medications and airway equipment available in your practice.

Based on this information, please answer the following questions:

13. **Intravenous access (IV) access**: In a patient similar to the one described in the above scenario (Scenario 2, CHF), how often would you **insert the following PRIOR** to intubation?

    Please remember that **the patient has no IV access** at the present time.

	Never	Rarely	Sometimes	Often	Always
Single peripheral IV	○	○	○	○	○
Multiple peripheral IV's	○	○	○	○	○
Central venous catheter	○	○	○	○	○
Arterial catheter	○	○	○	○	○

14. **IV fluid administration before emergent endotracheal intubation:** In a patient similar to the one described in the above scenario (Scenario 2, CHF), please indicate what class of IV fluid (if any) that you would administer **PRIOR** to intubation.

    Please remember that **this patient has not received any IV fluids** prior to this time.

	Never	Rarely	Sometimes	Often	Always
None	○	○	○	○	○
Crystalloid (0.9% saline/Lactated Ringers)	○	○	○	○	○
5% albumin	○	○	○	○	○
25% albumin	○	○	○	○	○
Packed Red Blood Cells	○	○	○	○	○
Synthetic colloid (HES)	○	○	○	○	○

15. **IV FLUID Volume:** In a patient similar to the one described in the above scenario (Scenario 2, CHF), please indicate the **approximate volume of IV fluid** that you would normally administer **PRIOR** to intubation.

Please base your answer on the **most preferred IV fluid as indicated in the question above**.

-

	Never	Rarely	Sometimes	Often	Always
No IV fluid administration	○	○	○	○	○
<500 ml	○	○	○	○	○
500-999 ml	○	○	○	○	○
1000-1499 ml	○	○	○	○	○
1500-1999 ml	○	○	○	○	○
>2000 ml	○	○	○	○	○

16. **Vasopressor Administration:** In a patient similar to the one described in the above scenario (CHF), please indicate which (if any) of the following that you would normally administer (**either bolus or infusion**) **PRIOR** to intubation.

Please note that this patient has not reveived any interventions prior to this time. His vital signs are HR 120 bpm, RR 32/min, BP 100/56 mmHg, SaO2 90% (on FiO2 100%) and temperature 36.9 C.

	Never	Rarely	Sometimes	Often	Always
phenylephrine	○	○	○	○	○
ephedrine	○	○	○	○	○
dopamine	○	○	○	○	○
norepinephrine	○	○	○	○	○
epinephrine	○	○	○	○	○
dobutamine	○	○	○	○	○
vasopressin	○	○	○	○	○

17. In patients similar to Scenario 2 (CHF), please indicate how often you would **administer a peripheral vasopressor prior to intubation** (assuming the patient has no central venous access):

	Never	Rarely	Sometimes	Often	Always
ADMINISTER a vasopressor via a PERIPHERAL IV before intubation	○	○	○	○	○
WAIT to administer a vasopressor until after a	○	○	○	○	○

CENTRAL VENOUS CATHETER is inserted

A Survey of Emergent Endotracheal Intubation in Critically Ill Patients in Canada

18. In your practice, how often would you use the following medications to facilitate intubation in patients similar to the patient described in the above scenario (Scenario 2, CHF)?

	Never	Rarely	Sometimes	Often	Always
midazolam	○	○	○	○	○
other benzodiazepine	○	○	○	○	○
fentanyl	○	○	○	○	○
other opioid	○	○	○	○	○
etomidate	○	○	○	○	○
propofol	○	○	○	○	○
ketamine	○	○	○	○	○
succinylcholine	○	○	○	○	○
rocuronium	○	○	○	○	○

19. **Primary intubation strategy**: In a patient similar to the one described in the above scenario (scenario 2, CHF), **how often would you use the following devices/procedures** to intubate this patient?
-

	Never	Rarely	Sometimes	Often	Always
Curved blade (Macintosh) direct laryngoscope	○	○	○	○	○
Straight blade (Miller) direct laryngoscope	○	○	○	○	○
Bougie assisted direct laryngoscope	○	○	○	○	○
Video laryngoscope (ie. glidescope)	○	○	○	○	○
Other indirect devices (ie. ILMA, optical stylet, airtrac, lightwand, etc.)	○	○	○	○	○
Flexible bronchoscope	○	○	○	○	○
Transtracheal jet ventilation	○	○	○	○	○
Percutaneous wire guided cricothyrotomy	○	○	○	○	○
Open cricothyrotomy	○	○	○	○	○

Tracheostomy	○	○	○	○	○

20. **Secondary/back up intubation strategy**: In the event that **you could not intubate** a patient similar to the paient in Scenario 2 (CHF), how often would you perform the following?

-

	Never	Rarely	Sometimes	Often	Always
Extraglottic Device (LMA, King LT, etc)	○	○	○	○	○
Trastracheal Jet Ventilation	○	○	○	○	○
Percutaneous Wire Guided Cricothyrotomy	○	○	○	○	○
Open Cricothyrotomy	○	○	○	○	○
Tracheostomy	○	○	○	○	○

A Survey of Emergent Endotracheal Intubation in Critically Ill Patients in Canada

After successful intubation without complication or difficulty, the patient's blood pressure is reduced from a pre-intubation blood pressure of 100/56 mmHg.
For the purposes of the following questions, please assume that the patient's chronic "hypertension" is adequately controlled with outpatient medications, and that he currently has no evidence of other adverse events (ie: SaO2 100%, no cardiac ischemia).

Based on this information, please answer the following information.

21. When considering blood pressure reductions after intubation in patients similar to the patient in scenario 2 (CHF), what is **the threshold that you would you consider to be "unstable" and actively intervene?**
Select at least 1 and no more than 1.

- ☐ Systolic Blood Pressure <100
- ☐ Systolic Blood Pressure <90
- ☐ Systolic Blood Pressure <80
- ☐ Systolic Blood Pressure <70
- ☐ Systolic Blood Pressure <60
- ☐ Other, please specify

22. When considering blood pressure reductions after intubation in patients similar to the patient in scenario 2 (CHF), what is **the Mean Arterial Pressure (MAP) threshold that you would actively intervene?**
Select at least 1 and no more than 1.

- ☐ Mean Arterial Pressure <80
- ☐ Mean Arterial Pressure <70
- ☐ Mean Arterial Pressure <60
- ☐ Mean Arterial Pressure <50
- ☐ Mean Arterial Pressure <40
- ☐ Other, please specify

23. Please assume that your **patient meets your threshold for requiring intervention** for hemodynamic instability after emergent intubation. **How long does a patient similar to the patient in scenario 2 (CHF) have to be hemodynamically unstable before you intervene**?

Select at least 1 and no more than 1.

- [ ] I would treat hemodynamic instability immediately.
- [ ] 1-2 min; I would treat hemodynamic instaility if it has been present for >2 min.
- [ ] 2-4 min; I would treat hemodynamic instability if it has been present for >4 min.
- [ ] 4-6 min; I would treat hemodynamic instability if it has been present for >6 min.
- [ ] 6-8 min; I would treat hemodynamic instability if it has been present >8 min.
- [ ] >10 min; I would treat hemodynamic instability if it has been present >10 min.
- [ ] Never; I would not treat hemodynamic instability.
- [ ] Other, please specify

24. Assuming that a patient similar to the patient in scenario 2 (CHF) **meets your blood pressure threshold and duration criteria for post-intubation hemodynamic instability**, how would you **manage the patient's hemdynamic instability**?

Please assume that his low blood pressure is the only issue at this time (Oxygen saturation 100%, no evidence of cardiac ischemia or other end-organ dysfunction).

-

	Never	Rarely	Sometimes	Often	Always
Crystalloid (0.9% saline or Lacteted Ringer's) IV fluid administration	○	○	○	○	○
Albumin IV administration	○	○	○	○	○
Synthetic colloid (HES) IV fluid administration	○	○	○	○	○
vasopressor administration via a peripheral IV (if no central catheter)	○	○	○	○	○
Vasopressor administration via a central catheter	○	○	○	○	○
I would not treat post-intubation hemodynamic instability	○	○	○	○	○

A Survey of Emergent Endotracheal Intubation in Critically Ill Patients in Canada

Scenario 3: Trauma

You are asked to see a 29-year-old male in the Emergency Department who was involved in a motor vehicle collision. He was a belted passenger of a single vehicle crash in which the driver was killed. He has abrasions on his head, chest and abdomen and is immobilized with a cervical spine collar and backboard.

He was previously healthy and his weight is 90Kg. His vital signs are HR 120 bpm, RR 32/min, BP 100/56 mmHg, SaO2 90% (on FiO2 100%) and temperature 36.9 C. His GCS is 6.

A bedside ultrasound of his abdomen reveals free fluid, and you feel that he is actively bleeding from an intra-abdominal source, and that **he requires immediate intubation to facilitate further management.**

He has no predictors of a difficult airway other than the cervical immobilization. He has no IV access at this point, and has received no other interventions prior to your arrival. You have the aid of a skilled acute care nurse in addition to an airway cart containing the usual medications and airway equipment available in your practice.

Based on this information, please answer the following questions:

25. **Intravenous access (IV) access**: In a patient similar to the one described in the above scenario (Scenario 3, Trauma), how often would you **insert the following PRIOR** to intubation?

Please remember that **the patient has no IV access** at the present time.

	Never	Rarely	Sometimes	Often	Always
Single peripheral IV					
Multiple peripheral IV's					
Central venous catheter					
Arterial catheter					

26. **IV fluid administration before emergent endotracheal intubation:** In a patient similar to the one described in the above scenario (Scenario 3, trauma), please indicate what class of IV fluid (if any) that you would administer **PRIOR** to intubation.

Please remember that **this patient has not received any IV fluids** prior to this time.

	Never	Rarely	Sometimes	Often	Always
None					
Crystalloid (0.9% saline/Lactated Ringers)					
5% albumin					
25% albumin					
Packed Red Blood Cells					
Synthetic colloid (HES)					

27. **IV FLUID Volume:** In a patient similar to the one described in the above scenario (Scenario 3, trauma), please indicate the **approximate volume of IV fluid** that you would normally administer **PRIOR** to intubation.

Please base your answer on the **most preferred IV fluid as indicated in the question above**.

-

	Never	Rarely	Sometimes	Often	Always
No IV fluid administration					
<500 ml					
500-999 ml					
1000-1499 ml					
1500-1999 ml					
>2000 ml					

28. **Vasopressor Administration:** In a patient similar to the one described in the above scenario (Scenario 3, trauma), please indicate which (if any) of the following that you would normally administer (**either bolus or infusion**) **PRIOR** to intubation.

Please note that this patient has not reveived any interventions prior to this time. His vital signs are HR 120 bpm, RR 32/min, BP 100/56 mmHg, SaO2 90% (on FiO2 100%) and temperature 38.9 C.

	Never	Rarely	Sometimes	Often	Always
phenylephrine	○	○	○	○	○
ephedrine	○	○	○	○	○
dopamine	○	○	○	○	○
norepinephrine	○	○	○	○	○
epinephrine	○	○	○	○	○
dobutamine	○	○	○	○	○
vasopressin	○	○	○	○	○

29. In patients similar to Scenario 3 (Trauma), please indicate how often you would **administer a peripheral vasopressor prior to intubation** (assuming the patient has no central venous access):

	Never	Rarely	Sometimes	Often	Always
ADMINISTER a vasopressor via a PERIPHERAL IV before intubation	○	○	○	○	○
WAIT to administer a vasopressor until after a CENTRAL VENOUS CATHETER is inserted	○	○	○	○	○

A Survey of Emergent Endotracheal Intubation in Critically Ill Patients in Canada

30. In your practice, how often would you use the following medications to facilitate intubation in patients similar to the patient described in the above scenario (Scenario 3, Trauma)?

	Never	Rarely	Sometimes	Often	Always
midazolam	○	○	○	○	○
other benzodiazepine	○	○	○	○	○
fentanyl	○	○	○	○	○
other opioid	○	○	○	○	○
etomidate	○	○	○	○	○
propofol	○	○	○	○	○
ketamine	○	○	○	○	○

succinylcholine	○	○	○	○	○
rocuronium	○	○	○	○	○

31. **Primary intubation strategy**: Based on a patient similar to the one described in the above scenario (Scenario 3, trauma), **how often would you use the following devices/procedures** to intubate this patient?
-

	Never	Rarely	Sometimes	Often	Always
Curved blade (Macintosh) direct laryngoscope	○	○	○	○	○
Straight blade (Miller) direct laryngoscope	○	○	○	○	○
Bougie assisted direct laryngoscope	○	○	○	○	○
Video laryngoscope (ie. glidescope)	○	○	○	○	○
Other indirect devices (ie. ILMA, optical stylet, airtrac, lightwand, etc.)	○	○	○	○	○
Flexible bronchoscope	○	○	○	○	○
Transtracheal jet ventilation	○	○	○	○	○
Percutaneous wire guided cricothyrotomy	○	○	○	○	○
Open cricothyrotomy	○	○	○	○	○
Tracheostomy	○	○	○	○	○

32. **Secondary/back up intubation strategy**: In the event that **you could not intubate** a patient similar to the one described in the above scenario (Scenario 3, trauma), how often would you perform the following?

-

	Never	Rarely	Sometimes	Often	Always
Extraglottic Device (LMA, King LT, etc)	○	○	○	○	○
Trastracheal Jet Ventilation	○	○	○	○	○
Percutaneous Wire Guided Cricothyrotomy	○	○	○	○	○
Open Cricothyrotomy	○	○	○	○	○
Tracheostomy	○	○	○	○	○

A Survey of Emergent Endotracheal Intubation in Critically Ill Patients in

Canada

After successful intubation without complication or difficulty, the patient's blood pressure is reduced from a pre-intubation blood pressure of 100/56 mmHg.
For the purposes of the following questions, please assume that he currently has no evidence of any adverse events (ie: SaO2 100%, no cardiac ischemia).

Based on this information, please answer the following information.

33. When considering blood pressure reductions after intubation in patients similar to the patient in scenario 3 (Trauma), what is **the threshold that you would you consider to be "unstable" and actively intervene?**
Select at least 1 and no more than 1.

☐ Systolic Blood Pressure <100
☐ Systolic Blood Pressure <90
☐ Systolic Blood Pressure <80
☐ Systolic Blood Pressure <70
☐ Systolic Blood Pressure <60
☐ Other, please specify

34. When considering blood pressure reductions after intubation in patients similar to the patient in scenario 3, what is **the Mean Arterial Pressure (MAP) threshold that you would actively intervene?**
Select at least 1 and no more than 1.

☐ Mean Arterial Pressure <80
☐ Mean Arterial Pressure <70
☐ Mean Arterial Pressure <60
☐ Mean Arterial Pressure <50
☐ Mean Arterial Pressure <40
☐ Other, please specify

35. Please assume that you **patient meets your threshold for requiring intervention** for hemodynamic instability after emergent intubation. **How long does a patient similar to the patient in scenario 3 (Trauma) have to be hemodynamically unstable before you intervene**?

Select at least 1 and no more than 1.

☐ I would treat hemodynamic instability immediately.
☐ 1-2 min; I would treat hemodynamic instaility if it has been present for >2 min.
☐ 2-4 min; I would treat hemodynamic instability if it has been present for >4 min.
☐ 4-6 min; I would treat hemodynamic instability if it has been present for >6 min.
☐ 6-8 min; I would treat hemodynamic instability if it has been present >8 min.
☐ >10 min; I would treat hemodynamic instability if it has been present >10 min.
☐ Never; I would not treat hemodynamic instability.
☐ Other, please specify

36. Assuming that a patient similar to the patient in Scenario 3 (Trauma) **meets your blood pressure threshold and duration criteria for post-intubation hemodynamic instability**, how would you **manage the patient's hemdynamic instability**?

Please assume that his low blood pressure is the only issue at this time (Oxygen saturation 100%, no evidence of cardiac ischemia or other end-organ dysfunction).

-

	Never	Rarely	Sometimes	Often	Always

Crystalloid (0.9% saline or Lacteted Ringer's) IV fluid administration	◯	◯	◯	◯	◯
Albumin IV administration	◯	◯	◯	◯	◯
Synthetic colloid (HES) IV fluid administration	◯	◯	◯	◯	◯
vasopressor administration via a peripheral IV (if no central catheter)	◯	◯	◯	◯	◯
Vasopressor administration via a central catheter	◯	◯	◯	◯	◯
I would not treat post-intubation hemodynamic instability	◯	◯	◯	◯	◯

A Survey of Emergent Endotracheal Intubation in Critically Ill Patients in Canada

Investigations of Emergent Endotracheal Intubaton

We would like to ask your opinions on the importance of investigations of emergent endotracheal intubation and your willingness to participate.

37. Do you feel that clinical studies in patients requiring emergent endotracheal intubation are **warranted at this time**?

- ◯ Strongly Agree
- ◯ Agree
- ◯ Neutral
- ◯ Disagree
- ◯ Strongly Disagree

38. Would you be **willing to participate** in future clinical studies (including a randomized controlled trial) of IV fluid administration prior to intubation in patients requiring emergent endotracheal intubation?

- ◯ Strongly Agree
- ◯ Agree
- ◯ Neutral
- ◯ Disagree
- ◯ Strongly Disagree
- ◯ Other, please specify

39. Would you be **willing to participate** in future clinical studies (including a randomized controlled trial) of **vasopressor administration via central venous catheter** prior to intubation in patients requiring emergent endotracheal intubation?

- ◯ Strongly Agree
- ◯ Agree
- ◯ Neutral

- Disagree
- Strongly Disagree
- Other, please specify

40. Would you be **willing to participate** in future clinical studies (including a randomized controllled trial) of **vasopressor administration via peripheral venous catheter** prior to intubation in patients requiring emergent endotracheal intubation.

- Strongly Agree
- Agree
- Neutral
- Disagree
- Strongly Disagree
- Other, please specify

A Survey of Emergent Endotracheal Intubation in Critically Ill Patients in Canada

DEMOGRAPHICS

Please note all responses are confidential.

41. In what province or territory do you currently practice?
Select at least 1 and no more than 1.

- Alberta
- British Columbia
- Manitoba
- New Brunswick
- Newfoundland/Labrador
- Northwest Territories
- Nova Scotia
- Nunavut
- Ontario
- Quebec
- Saskatchewan
- Yukon
- Other, please specify

42. What is your specialty?

- Emergency Medicine (FRCPC)
- Emergency Medicine (CCFP-EM)
- Emergency Medicine (CCFP or other)
- Family Medicine
- Internal Medicine
- Anesthesia
- General Surgery or a surgical sub-speciality
- Other, please specify

43. Have you completed a fellowship in Critcal Care Medicine?

- Yes
- No

44. Do you perform emergent endotracheal intubations as part of your current practice?

- yes
- no

45. How many years have you been in practice?
Select at least 1 and no more than 1.

- <1 year
- 1-5 years
- 6-10 years
- 11-20 years
- >20 years

46. In what type of hospital do you practice?*
Select at least 1 and no more than 1.

- Teaching (university affiliated)
- Community (no university affiliation)

A Survey of Emergent Endotracheal Intubation in Critically Ill Patients in Canada

Thank You!

47. Thank you very much for your time. By completeing this survey, you have helped advance care for criically ill patients requiring endotracheal intubation.
